# Using ESN-Smartphone Application to Maximize AIS Reperfusion Therapy in Alexandria Stroke Network: A Stroke Chain of Survival Organizational Model

**DOI:** 10.3389/fneur.2021.597717

**Published:** 2021-02-23

**Authors:** Ossama Yassin Mansour, Ismail Ramadan, Amer Elfatatry, Mohamed Hamdi, Ashraf Abudu, Tamer Hassan, Hany Eldeeb, Hazem Marouf, Mohamed Mogahed, Mohamed Farouk, Mohamed Abas, Mervat Hamed, Mohamed Afify, Tamer Abdallah, Osama Zaidat

**Affiliations:** ^1^Stroke Center, Semouha Emergency University Hospital, Alexandria, Egypt; ^2^Stroke Unit, Elhadara University Hospital, Alexandria, Egypt; ^3^Louran Comprehensive Stroke Center, Alexandria, Egypt; ^4^Mabaret Elasafra Hospital, Alexandria, Egypt; ^5^Shark el Madina Ministry of Health Hospital, Alexandria, Egypt; ^6^Department of Neurology, Damanhur Medical National Institute, Damanhur, Egypt; ^7^Elandalusia General Hospital, Alexandria, Egypt; ^8^Bon Secours Mercy Health System, Neuroscience Institute, St. Vincent Hospital, Toledo, OH, United States

**Keywords:** ESN-smartphone application, alexandria stroke network, reperfusion therapy, decision making, AIS, mechanical thrombectomy, thrombolysis

## Abstract

**Background:** In developing countries like Egypt, the clinical workflow of stroke management is poorly established due to the lack of awareness of the stroke patients concerning their need of therapeutic intervention and the poor identification of facilities equipped to treat stroke. Hence, establishing a stroke system of care in developing countries that can efficiently and rapidly triage patients to the appropriate reperfusion therapy center is imperative to improving stroke management and outcomes.

**Aims:** To evaluate a pilot experience in stroke hospital identification and expediting decision-making in AIS treatment through the Alexandria stroke network and Egyptian Stroke Network (ESN)-app.

**Methods:** Between 2017 and 2019, seven hospitals registered themselves on the AS-Network as pilot hospitals. The ESN-application was used to detect stroke type, tele-connect stroke teams and hospitals, track triage of patients to equipped facility in real time, and streamline stroke workflow. The quality of and time required for stroke management were compared between 84 patients with acute ischemic stroke (AIS) whose treatment involved the ESN-app and 276 patients whose treatment did not.

**Results:** During this pilot study, 360 AIS cases received reperfusion therapy, 84 of which were indicated by the ESN-app. The use of the application was associated with the significant drop in time metrics for the reperfusion AIS-patients (door-in-door-out time; 56 ± 34 min vs. 96 ± 45 min, door-to-groin puncture time; 50 ± 7 min vs. 120 ± 25 min, door-to-needle time; 55 ± 12 min vs. 78 ± 16 min with *p* < 0.0001). Its use was also associated with higher rates of excellent outcomes at the 90-day follow-up (without ESN-app vs. with ESN-app, 67.9 vs. 47.1%, *p* = 0.001) but no difference in 90-day mortality or symptomatic intracerebral hemorrhage (without ESN-app vs. with ESN-app, 9.5 vs. 11.2% and 4.8 vs. 5.1%, *p* > 0.05).

**Conclusion:** Our pilot experience demonstrated that the use of the ESN-app expedited the stroke treatment workflow and facilitated tele-connection between registered stroke facilities. Additionally, its use might be associated with achieving higher rates of excellent outcomes at 90 days, where a larger scale study is needed for more confirmation.

## Introduction

With an annual incidence of 270,000–960,000 (1), stroke is the second most frequent cause of death and the most frequent cause of disability in Egypt. In developing countries, many obstacles hinder the maximization of reperfusion therapy for acute ischemic stroke (AIS) (2). These include the limited public awareness of stroke symptoms; the delayed dispatch of emergency medical service (EMS), emergency transportation, and pre-hospital notification; the lack of clinical protocols and prehospital pathways; unrecognized stroke-ready facilities; poorly equipped emergency department and in-hospital stroke code activation; and the clear time-bound workflow for stroke care (3). No national program exists in Egypt for AIS management. Recently, AIS clinical scenarios were considered for partial reimbursement by the state (4), despite constant efforts to improve government understanding the cost of stroke care.

However, apart from efforts to improve stroke care in Egypt through legislation, providing timely AIS interventions to a larger proportion of the Egyptian population following the stroke event can be improved by streamlining stroke management following the dispatch of the EMS (5). Before their arrival at the hospital, EMS dispatchers play a key role in recognizing stroke symptoms, prioritizing the call, and initiating first response, who then transports the patient to and notifies the appropriate facility (6). Further, it is necessary to establish AIS-rapid triage protocols at emergency departments to ensure the immediate activation of the stroke team, fast throughput for rapid clinical evaluation, the efficient performance of non-contrast head computerized tomography, and prompt treatment decisions. Only by having all these elements in place can the delay in initiating AIS therapy be avoided, the initial phase of the chain of recovery be completed, and the clinical outcomes of stroke patients be improved (7, 8). To achieve these results, we initiated the Alexandria Stroke Network (ASN) project to organize a stroke service chain of survival in the region of Alexandria. Specifically, ASN uses mobile smartphone technology in the triage and management of patients with AIS to streamline the management of stroke patients (9). We herein describe our pilot experience with connecting an ESN smartphone application to the Alexandria stroke network and show if any effect of using ESN-app on stroke workflow via measuring impact on different time metrics.

## Methods and Patients

The second-largest city in Egypt, Alexandria extends about 32 km along the Mediterranean coast and features a total area of 2,679 km^2^, a total population of 5.2 million, and a population density of 1,900/km^2^. As the city is considered a hub for other districts, including the Elbehera, Matrouh, and Kafer-elshiekh governorates, the city's population rises to virtually 11 million (10).

### Forming the Alexandria Stroke Network

Neurology training hospitals and hospitals that provide acute stroke care in Alexandria and the surrounding areas were invited to register their hospital's information *on the registry website* (www.strokeregistry.eg). The information requested at the time of hospital registration included: [1] availability of providers in the disciplines of neurology, neurosurgery, emergency, and neuroradiology, as well as neurointerventional national board certified specialists; [2] availability of specific treatments, including IV thrombolysis, mechanical thrombectomy, and neurosurgery; [3] presence of a dedicated stroke unit, or stroke-assigned beds in the intensive care unit; [4] the availability of CT and/or MRI and their hours of operation; and [5] the hospital's address, emergency telephone number, and hours of operation. Upon receiving a hospital's submission to register on the web page, an administrator reviewed the information provided and approved their registration to the site. Based on the capability of each hospital to fulfill the aforementioned five stroke-care service bundles (Supplement), as determined from the information submitted, each hospital was assigned a color from a 6-tier color-coded grading system.

Seven hospitals were registered and approved at the time of the submission of this manuscript. The seven participating hospitals are geographically distributed across the city. Each entered AIS data into an official web-based stroke registry of the Alexandria stroke network project (SECRET-registry). A unique account through web-based data entry interface for the cloud based server of the SECRET-registry was established for each approved hospital. Typically, a neurologist from a given hospital is granted access to the registry to manage and/or submit cases and answer database queries. Additionally, the monitoring of performance quality metrics was possible through a tool developed with PHP (recursive **acronym for PHP**: Hypertext Preprocessor) and java script, allowing each hospital to compare its metrics against the common pool indicating city-wide performance. Data protection on the registry server is maintained through Cloud security technologies.

### Technical Application Specifications

An Egyptian stroke network application (ESN-app) for smartphones was developed to organize the stroke service chain of survival in Egypt, starting with Alexandria as a model. The ESN-app was developed for multi-platform smartphones, including iPhone™ (https://mena-sino.live/AppleStore/ESN-app) and Android™ (https://mena-sino.live/Playstore/ESN-app), using the iPhone software development toolkit (SDK 3.0, Apple Inc., Cupertino, CA, USA) and the Android SDK (SDK r20.0.3) (11). Map data for the application were adopted from the open map (Alexandria map API v3, Alexandria Comp., Alexandria, Egypt).

The ESN-app was developed as a hybrid app that concurrently provides access to both case data entry and the map data. This feature enables users to connect to the web-based SECRET registry with pre-populated, continually updated, and accurate map information. A distance-calculation algorithm was adapted from the Haversine formula.

### Functions of the ESN Application

**Figure 1:**
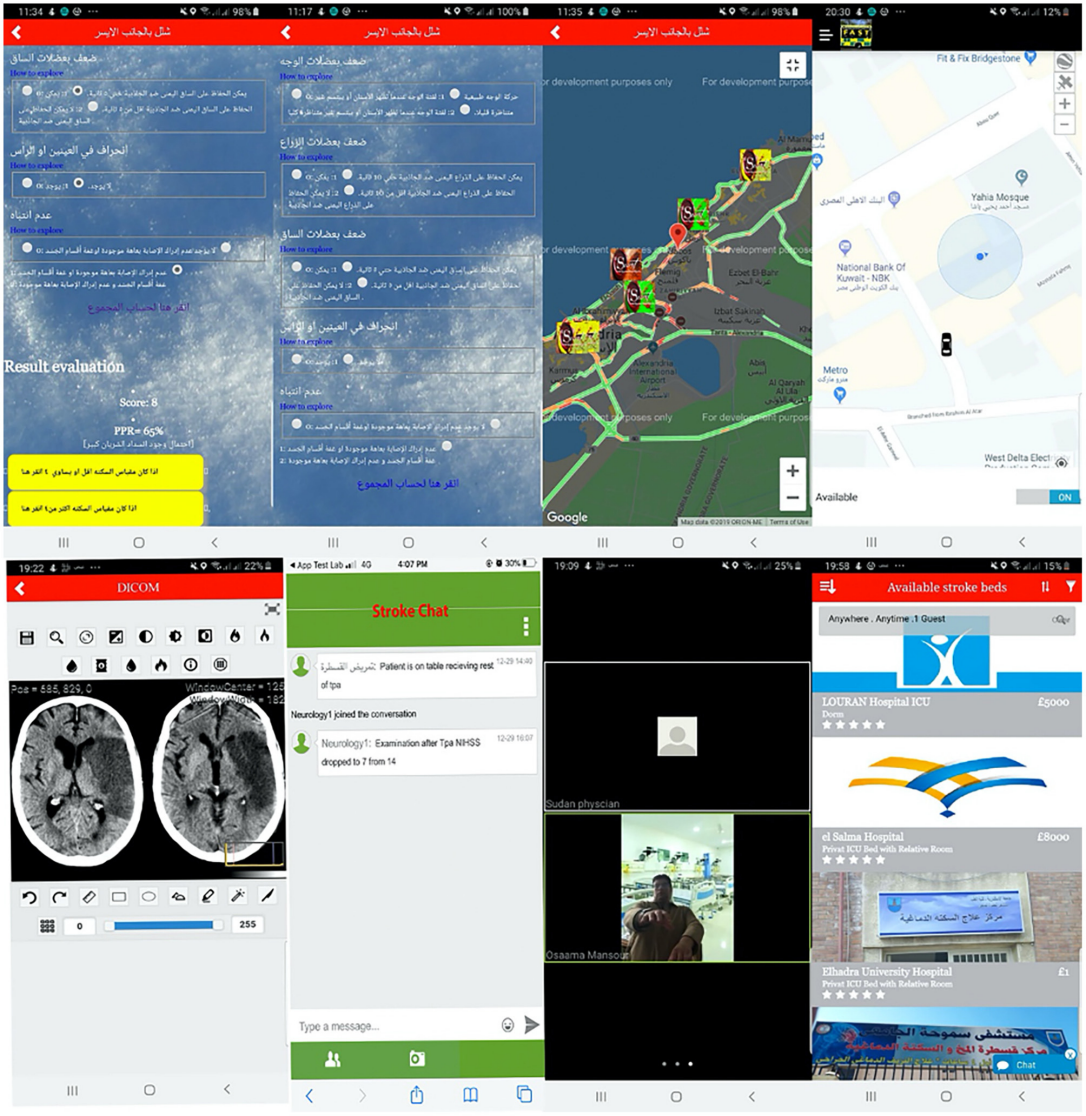
Egyptian stroke network (ESN)-application functions.

The ESN-app includes a large vessel occlusion (LVO) stroke-screening tool and real-time information on nearby reperfusion therapy-capable hospitals. The ESN-app LVO screening tool is informed by the results of the Arabic interfaced RACE-based LVO stroke detection tool. Specifically, it consecutively displays five questions that assess the presence of facial palsy, upper and lower limb weakness, gaze movement, and speech and gnostic disturbance. The user can select from possible answers whose answers indicate whether he or she may be having a stroke. This interface also displays the total score and a predictive percentage for LVO and two yellow buttons signaling either a high LVO prediction or Low LVO prediction (Figure 1). The selection of either prompts a pop-up window showing a GPS map with nearby hospitals coded according to their level of stroke care capability.

Upon an affirmative decision to initiate transfer, the stroke team (i.e., stroke neurology and neurosurgery services, anesthesia team, nurse, neuro-interventional technologist, and neuro-intensive care unit physician) is notified via automated notification page. Using the ESN-app, the entire stroke team can follow the ambulance en route to the hospital – from the pickup point to the drop-off location (Figure 1). The ESN-app allows for real-time, secure telecommunication between the members of the stroke team and the recording of events in the stroke management workflow with automated timestamps. Some timestamped events can be recorded as a log page that includes the date and time of onset, presentation at first hospital, arrival at treating facility, IV-thrombolysis door-to-needle (DTN) time, neuroimaging completion, arrival to the angiography suite, groin puncture, first pass, and final angio-run.

All timestamps were manually input into the app by one of the team members (usually the neurology physician on call) and become visible to all team members via timestamped notifications on their smartphone ESN-app (Figure 1). The application also features a secure, two-way video system that allows for video-based interhospital teleconsultation as needed to maximize IV-thrombolysis.

### Outcomes

We compared the metrics and outcomes between ESN-app- and non-ESN-app-facilitated AIS triage, including door-to-needle (DTN) times, LVO detection rates, AIS transfer time metrics (door-in-door-out [DIDO] time, ambulance-call-to-ER time), and door-to-groin puncture time. In addition, the rate of functional independence at 90 days, as assessed with the modified Rankin's Scale (mRS), was compared between the two groups.

### Statistical Analysis

The data for ESN-app triage and non-ESN-app triage were compared using Student's *t*-test for normally distributed continuous variables. The Chi-square and Fisher's exact tests were employed to compare proportions between the two groups.

## Results

### Patient-Care Quality

The seven participating hospitals included one comprehensive stroke center the functioned as a hub and six satellite hospitals. Each hospital entered data into the AIS SECRET-registry. During the study period, 1,848 patients with suspected AIS were entered into the registry (Supplement) by the seven participating hospitals at an average of 20 cases per month (Table 1).

**Table 1:** Patient data uploaded by participating hospitals.

**Patients received full reperfusion workflow (n)**	**App - patients (*****n*** **=** **84)**		**Non-app - patients (*****n*** **=** **276)**	
	**App assisted IV thrombolysis (*n* = 64)**	**App assisted MT (*n* = 36)**	**Non-app assisted IV thrombolysis (*n* = 123)**	**Non-app assisted MT (*n* = 153)**
Patients received one modality (either IV-rtpa or MT code)	(*n* = 48 patients received full dose IV-tpa only)	(*n* = 16 patients received MT only)	(*n* = 100 patients full dose IV-rtpa only)	(*n* = 153 patients only MT (#n=23 of them received MT after half dose IV tpa)
Patients received 2 modalities (Bridging Therapy)	(*n* = 20 patients received additional MT after full dose IV-rtpa *)		(*n* = 23 patients received additional MT after full dose IV-rtpa)	
**Breakdown of reperfusion procedures by hospitals**				
1 (loran hospital)	30	31	4	145
2 (Smouha university hospital)	11	2	60	4
3 (Narmean university hospital)	8	0	34	0
4 (Damanhur educational hospital)	5	0	0	0
5 (Shark el-medina MOH)	4	0	0	0
6 (Mabret el asafra hospital)	5	0	9	0
7 (Andalusia medical group hospitals)	5	3	16	4

*IV-tpa, intravenous tissue plasminogen activator; MT, mechanical thrombectomy*.

**Figure 2:**
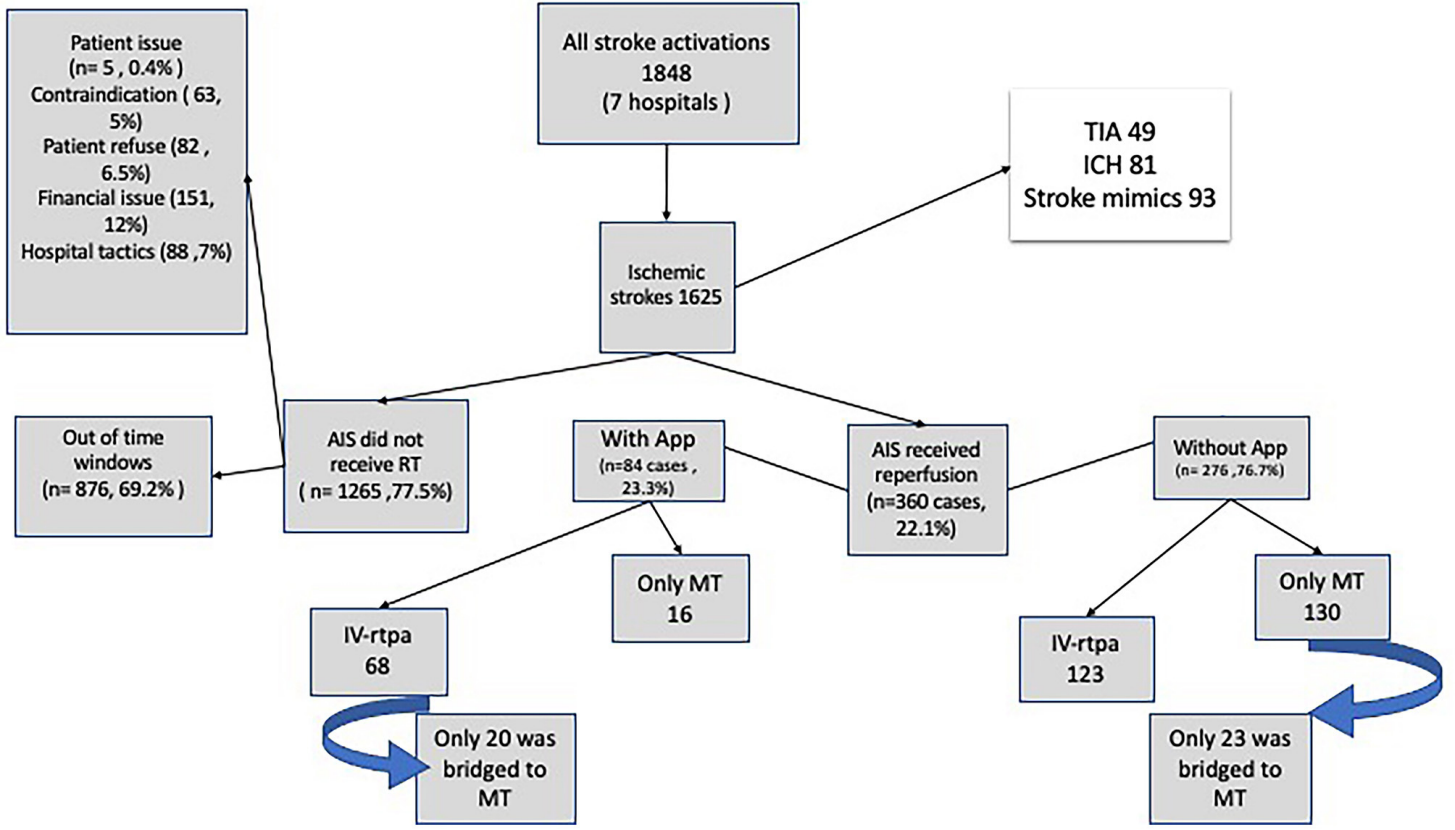
Flowchart of the patients included in the study. ICH indicates intracranial hemorrhage; TIA, transient ischemic attack; and tPA, tissue-type plasminogen activator; MT, Mechanical thrombectomy; IA, Intraarterial; AIS, acute ischemic stroke.

Of the 1,848 patients, 1,626 presented with ischemic stroke, 49 with transient ischemic attack, 81 with hemorrhagic stroke, and 93 with stroke mimics or other cerebrovascular diseases. Stroke code was initiated in 360 cases: 276 AIS reperfusion decisions (ARDs) were made without the assistance of the ESN-app, while 84 ARDs were made with reference to the ESN-app (Figure 2). In all 84 cases, the patients were initially assessed by neurology residents (excepting 12 cases at the hub hospital) via ESN-app based teleconsultation with a stroke neurologist.

### Baseline Characteristics of the Patients

The baseline characteristics of the 84 patients with AIS (mean age, 60 ± 5 years; 52% male; 30% cardioembolic strokes) whose treatment involved ESN-app activation are presented in Table 2. The initial mean NIHSS scores were numerically higher in the ESN-app-facilitated consultation group than in the non-ESN-app/telephonic consultation group: 14.5 ± 2.5 vs. 10.4 ± 3.6, respectively (*p* < 0.0001, *t* = 9.74.)

**Table 2:** Characteristics of patients entered into the registry.

**All pilot hospitals**	**App patients (*n* = 84)**	**NON app patients (*n* = 276)**	***P*-value**
Males	44 (52.3%)	149 (54%)	0.8
LVO	36 (42.8%)	153 (55.4%)	<0.05
Mean age	60 ± 5 y	63 ± 2 y	<0.0001
Mean NIHSS	14.5 ± 2.5	10.4 ± 3.6	<0.0001
Recurrent stroke/TIA	21 (25%)	60 (21.7%)	0.6
**Stroke subtype (TOAST)**			
Cardioembolic, n (%)	28 (33.3%)	87 (31.5%)	0.78
Large vessel athrosclerosis, n (%)	26 (30.9%)	102 (37%)	0.36
Lacunar, n (%)	14 (16.6%)	43 (15.6%)	0.86
Mortality at 3 mo, n (%)	8 (9.5%)	31 (11.2%)	0.84
Functional independence (mRS <3) at 3 mo, n (%)	57 (67.9%)	130 (47.1%)	0.0011
sICH	4 (4.8%)	14 (5.1%)	> 0.05

*LVO, large vessel occlusion; NIHSS, National Institute of Health Stroke Scale; TIA, transient ischemic attack; TOAST, Trial of **Org** 10172 in Acute Stroke Treatment (stroke classification); sICH, symptomatic intracerebral hemorrhage*.

The ESN-app was used in the 84 cases to assist in the following: [1] intravenous tissue plasminogen activator (IV-tPA) full dose administration at the remote satellite hospital (*n* = 56 cases) or in the hub hospital (*n* = 12) when no stroke expertise was available; [2] the triage and transfer of patients with LVO stroke to a mechanical thrombectomy (MT)-ready hospital (*n* = 36 cases); [3] the mobile tracking of and telecommunication with the transporting ambulance (*n* = 84 cases); and [4] streamlining the intrahospital stroke protocol workflow (Supplement) by documenting each step in the treatment of the AIS patients, from stroke onset to the completion of the reperfusion treatment, on the pre-notification page.

### Outcomes of ESN-App vs. Non-ESN App AIS Triage

The iv-tPA mean DTN time was significantly shorter for the 56 patients whose treatment at the satellite hospitals involved the ESN-app than for the 105 patients whose treatment did not (41 ± 4 min vs. 62 ± 14 min, *p* < 0.05). There was no gain in time in the 12 cases in which IV-thrombolysis was performed at the hub hospitals involved the ESN-app than for the 18 patients whose treatment did not (mean DTN time, 47 ± 8 min vs. 44 ± 12 min, *p* < 0.05). The LVO detection was significantly higher in the ESN-app triaged group than in the non-ESN-triaged group: 30/31 (96.7%) of the confirmed LVO vs. 23/47 (48.9%), respectively (*p* < 0.0001).

Time metrics (door-in-door-out [DIDO] time, ambulance-call-to-ER time, DTN time, and door-to-groin puncture [DTG] time) were significantly shorter among the patients who were managed and triaged with the ESN-app than among those who were not (56 ± 34 min vs. 96 ± 45 min, 45 ± 5 min vs. 98 ± 20 min, 55 ± 12 min vs. 78 ± 16 min, and 50 ± 7 min vs. 120 ± 25 min, respectively) (Table 3).

**Table 3:** Type of reperfusion therapy and time metrics in treatment workflow.

**Process of hospital care**	**App triaged patients**		**Non-App triaged patients**		***P*-value**
	***n***	**%**	***n***	**%**	
In ischaemic stroke, patients received intravenous thrombolysis (tPA)	68/84	81%	123/276	44.6%	<0.0001
LVO Patient transferred from another hospital within 6 h window	31/36	86.1%	47/153	30.7%	<0.0001
Confirmed LVO (transferred) within 6 h	30/36	(96.7%)	23 /47	(48.9%)	0.0001
Received MT (transferred) within 6 h	28/30	(93.3%)	16/23	(69.6%)	0.03
Door to needle time	55 ± 12 min		78 ± 16 min		<0.0001
Door in- door out time	56 ± 34 min		96 ± 45 min		<0.0001
Door to groin time	50 ± 7 min		120 ± 25 min		<0.0001
Ambulance to ER time	45 ± 5 min		98 ± 20min		<0.0001
Time to image	14 ± 4 min		23 ± 9 min		<0.0001

### Clinical Outcomes

Significantly higher functional independence rates (mRS 0-2 at 90 days follow up) were achieved for patients with AIS for whom treatment decision making was informed by the ESN-app (57 [67.9%] vs. 130 [47.1%], respectively; *p* = 0.0011).

The ESN-app secure text messaging communication feature facilitated the rapid delegation of tasks among the entire stroke care team and ensured the confirmation of their completion. This feature allowed for the detection of 123 drifts (deviations from the SOPs) and was instantaneously corrected during the stroke code: 72 (58.5%) were delayed while moving to next step of the workflow, 52 of (42.2%) dropped step/s of the stroke- code workflow, 18 (14.6%) resulted from the delay in obtaining consent for MT, and 27 (21.1%) were due to the delay in reporting clinical data, such as NIHSS, or uploading the radiological images.

The ESN-app successfully tracked all time stamps of the workflow. Continuous tracking of the patients' locations was achieved through a GPS chip connected to the seven registered ambulance cars (property of the registered hospitals that previously approved the registration of their ambulances into the system) in the 84 cases triaged by the ESN-app. The team arrival time was timestamped on the notification page in all cases. The stroke team arrival time was faster in the ESN-app triage group (*n* = 84) than in the non ESN-app triage group (*n* = 276; 15.05 ± 4.96 min vs. 25.03 min ± 8.66 min, respectively; *p* < 0.0001).

### Ascertainment and Competence of Data Entry

In the SECRET registry, the collection of all mandatory variables, including the patient background characteristics and processes of care variables, during acute triage was complete. Matching registry data to hospital discharge data, the results revealed high levels of completeness and accuracy in the records of the patients who were triaged with the application when compared to those entered in the registry without using the application.

### Team Satisfaction and Futuristic Enthusiasm

At the end of this pilot study, the 70 users of the ESN-app who were involved in this pilot study (including physicians and paramedics) were asked to answer brief questionnaires (Supplement): 96% of the users were satisfied with the reliability of the ESN-app, and 91% favored the use of the ESN-app to decrease patient downtime waiting. Using the Net Promoter Score (NPS) to assess the users' experiences and the degree of enthusiasm for the continued use of the ESN-app, only 3.13% were found to not show enthusiasm; the enthusiastic use rate was 56%.

## Discussion

The current study tested the efficacy and usability of our ESN-app to connect registered hospitals in the Alexandria stroke network, streamline the pre-hospital and intrahospital stroke workflow, and reduce the time consumed during the different steps of acute stroke management. Specifically, the app facilitated [1] AIS patient detection and treatment decision making at initial remote facility to improve DIDO time, [2] directing transportation in the prehospital stage to reduce transit time, and [3] intrahospital workflow to reduce door-to-reperfusion (DRT) time.

Similarly, like in the other in GPS-based mobile applications for stroke response management, the GPS technology of the ESN-app is designed to be used in two ways: static and dynamic. The former helps to locate the nearest treatment center, while the latter allows for the instantaneous tracking of the patient's location. The ESN-app thereby helps to coordinate immediate reperfusion therapy with other components of the AIS workflow (12, 13). The use of this technology maximized performance in the prehospital phase of EMS by reducing call-to-ambulance pickup and DIDO times in the group of patients transferred by the App (*p* < 0.05, *p* < 0.0001). This optimization of time efficiency be attributed to the ability of the ESN-app to continuously notify the stroke team of the patient's location, allowing the stroke team to time necessary preparations for the patient's arrival – including their own arrival at the hospital – rather than forcing them to remain idle on standby.

In current study, all of the LVO cases were transferred to the hub center with the assistance of the application and received reperfusion therapy within 6 h of onset: 31 patients received IV-tPA at the satellite hospital before their transfer, 28 of whom received MT. Hence, incorporating real-time prehospital data obtained via smartphone technology expedited and improved stroke treatment. These findings agree with those found in other developing countries by Andrew et al. (14).

One case was misdiagnosed by the App-RACE algorithm as LVO, and the patient's diagnosis was subsequently confirmed by CTA. Hence, the algorithm featured a sensitivity of 85.71% and specificity of 97.9%. This rate compares favorably with those reported in a recently published study (15). This scale has been validated and has been found to perform similarly relative to other pre-hospital LVO scales; i.e., it performs moderately well (16, 17).

A 24-min reduction in the DTN time for thrombolysis (from a median of 78 to 55 min) and a 71-min reduction in the DTG time for Thrombectomy (from a median of 120 to 50 min) was achieved with the use of the application. Hence, the positive impact of the application was comparable to the degree of improvement effected by more developed systems (18–20). Functional independence (mRS of < 3 at 90-day follow-up) was observed in 67.9% of the patients with AIS whose reperfusion was indicated by the application. Relative to the rate of 47.1% among the patients whose treatment was not informed by the application, the improvement in the attainment of functional independence indicates the important of reducing delay before reperfusion in improving outcomes and decreasing the complication rate associated with reperfusion (20, 21). Moreover, the application helped to maximize remote IV-thrombolysis by significantly reducing DTN (41 ± 4 min, *P* < 0.05).

Nogueira developed a well-designed, innovative FAST-ED scale-based smartphone application that accounts for the individual clinical characteristics of each patient to determine the patient's likelihood of requiring IV-tPA and/or EVT (22); this information is combined with real-world traffic data to direct the patient to the most appropriate hospital (primary stroke center for IV-tPA, and comprehensive stroke center for EVT). However, Nogueira application's design assumes an ideal world in which local politics and financial considerations are irrelevant. Such ideal circumstances differ greatly from the reality in Egypt, which is characterized by the absence of identified facilities and underutilization of EMS for stroke patients. Consequently, we created a micro-network comprised of several hospitals in Alexandria to facilitate their coordination through their registration into the system and to identify themselves according to their readiness to administer AIS reperfusion therapy (22). By connecting multiple facilities into a single network, our system allows each hospital to serve a large area and population.

A higher number of steps in a clinical workflow is associated with an increased incidence of medical errors. Reducing the number of hand-offs is likely associated with improving the efficiency and safety of stroke care (23, 24). Similarly, the current study found that the reduction of time of stroke workflow effected by the ESN-app was associated with the improvement of the efficiency and safety of the workflow. The presently observed sICH rate was 4.8%, which compared favorably with the rates of 5.6 and 3.7% reported by MT and IV thrombolysis studies, respectively (25, 26). Decreasing the number of hand-offs directly alleviates the fatigue of the treatment team – a relevant concern to the future expansion of MT (27) – by maximizing time efficiency. This has been shown by a Japanese study and is supported by the presently observed high satisfaction of our team concerning the capacity of the application to effectively organize the different steps of stroke care (28). Additionally, using the application reduced the duplication of tasks, such as contacting the patients' families for obtaining consent or payments for services. Similarly, the application minimized idle time due to changes in the patient condition during transportation – e.g., when patients are determined to not require reperfusion therapy – through real time tele-communication with the transportation team.

Although complete and accurate case ascertainment in clinical registries is essential to obtain valid and representative information, only a minority of cardiovascular disease–based registries report conducting case audits (29). However, an audit performed 3 months after our study found few absences in the data uploaded to the SECRET registry in cases triaged by the application.

The majority of the users of the application (95%) reported enthusiasm for the continued use of the application. We attribute this positive response to the utility of the application in improving the clinical workflow of stroke care. As the ESN-app described herein is the first generation of the ESN-smartphone application, the application can certainly be improved. The next version of the application will improve upon the questionnaires, application security, connection stability, automation of the timestamping of events, and the ease of submitting patient data to the SECRET stroke registry.

Study limitations include the non-randomized, retrospective registry design and small number size which may introduce some bias in the result interpretation.

## Conclusion

The pilot use of the ESN-app expedited stroke treatment workflow, as indicated by higher rates of excellent outcomes, and facilitated tele-connection between registered stroke facilities.

## Data Availability Statement

The original contributions presented in the study are included in the article/supplementary material, further inquiries can be directed to the corresponding author/s.

## Ethics Statement

The studies involving human participants were reviewed and approved by Alexandria university research ethical committee. Written informed consent for participation was not required for this study in accordance with the national legislation and the institutional requirements. Written informed consent was obtained from the individual(s) for the publication of any potentially identifiable images or data included in this article.

## Author Contributions

OM developed the theoretical formalism, performed the analytic calculations, and founded the idea and the network. OM and OZ contributed to the final version of the manuscript. OZ supervised the project. IR, MHamd, TH, HE, HM, MM, MF, MAb, MHame, MAf, and TA reviewed manuscript and participated in data analysis. AE, MF, and MAb have major role in data acquisition and analysis and writing the draft. All authors contributed to the article and approved the submitted version.

## Conflict of Interest

The authors declare that the research was conducted in the absence of any commercial or financial relationships that could be construed as a potential conflict of interest.
